# Microscopic Polyangiitis Mimicking Colitis: A Fatal Case of Fulminant Multi-organ Vasculitis

**DOI:** 10.7759/cureus.117963

**Published:** 2026-10-15

**Authors:** Amal Qaisar, Sumiyya Rashid, Simranjot Kaur

**Affiliations:** 1 Acute Medicine, University Hospitals of Leicester NHS Trust, Leicester, GBR

**Keywords:** acute colitis, anti-neutrophil cytoplasmic antibody (anca)-associated vasculitis (aav), localised vasculitis of the gastrointestinal tract, plasma exchange (plex), pulmonary haemorrhage, severe acidaemia, severe aki

## Abstract

Microscopic polyangiitis (MPA) is a systemic necrotising small vessel vasculitis that predominantly affects the kidneys and lungs. Gastrointestinal (GI) manifestations are less common and may mimic primary enteric disease, delaying recognition of the underlying systemic condition.

A 72-year-old woman presented with a one-week history of bloody diarrhoea, abdominal pain, vomiting, lower limb oedema, and purpura following recent ibuprofen use. Initial investigations demonstrated severe metabolic acidosis, stage 3 acute kidney injury (AKI), with an estimated glomerular filtration rate (eGFR) of 4 mL/min/1.73 m², active urinary sediment, and computed tomography (CT) evidence of extensive colitis with small bowel enteritis. Faecal testing for multiple enteric pathogens, including Shiga toxin-producing *Escherichia coli* (STEC), and testing for *Clostridioides difficile* were negative. Strongly positive myeloperoxidase anti-neutrophil cytoplasmic antibodies (MPO-ANCA; >221.9 IU/mL) supported a diagnosis of ANCA-associated vasculitis (AAV), while anti-glomerular basement membrane (anti-GBM) antibodies were negative. Despite prompt treatment with intravenous (IV) methylprednisolone, cyclophosphamide, therapeutic plasma exchange (PLEX), and intermittent haemodialysis, the patient developed fulminant diffuse alveolar haemorrhage (DAH), refractory hypoxaemia, consumptive coagulopathy, and severe acidaemia. She died on day 8 of admission. Renal biopsy histopathology, reported after death, demonstrated necrotising crescentic pauci-immune glomerulonephritis.

This case highlights the diagnostic challenges of MPO-ANCA-associated vasculitis presenting with severe gastrointestinal inflammation and bleeding alongside rapidly progressive renal disease. Although the renal findings confirmed ANCA-associated vasculitis, the underlying cause of the gastrointestinal abnormalities remained uncertain, with intestinal vasculitis and ischaemic injury secondary to hypoperfusion among the possible explanations. Recognition of systemic features in patients presenting with gastrointestinal illness and unexplained renal dysfunction is essential to facilitate timely investigation and treatment.

## Introduction

Anti-neutrophil cytoplasmic antibody (ANCA)-associated vasculitis (AAV) is a rare group of necrotising small-vessel vasculitides, with an estimated prevalence of approximately 200 cases per million population [1]. It comprises three main phenotypes: granulomatosis with polyangiitis (GPA), microscopic polyangiitis (MPA), and eosinophilic granulomatosis with polyangiitis (EGPA) [2]. ANCA serology is broadly divided into antibodies directed against proteinase 3 (PR3) and myeloperoxidase (MPO); GPA is more commonly associated with PR3-ANCA, whereas MPA is predominantly associated with MPO-ANCA [3].

MPA predominantly affects the renal and pulmonary systems, typically presenting with rapidly progressive glomerulonephritis and diffuse alveolar haemorrhage (DAH), although cutaneous purpura and other extra-renal manifestations may also occur [4]. Gastrointestinal (GI) involvement is less common and may manifest as colitis, intestinal ischaemia, bleeding, or perforation [5,6]. GI involvement may mimic more common primary gastrointestinal disorders, including infective or inflammatory colitis, potentially delaying recognition of the underlying systemic vasculitis. Severe gastrointestinal complications, including intestinal infarction, perforation, and major haemorrhage, have been associated with poor outcomes [5,6].

Despite advances in the management of AAV, including targeted immunosuppressive therapies and complement inhibition, fulminant multi-organ disease remains associated with substantial morbidity and mortality [1,2]. Early recognition of organ-threatening disease is therefore critical, particularly when seemingly unrelated manifestations occur concurrently. We report a patient with fulminant MPO-ANCA-associated vasculitis whose initial presentation was dominated by severe colitis, small bowel enteritis, and gastrointestinal bleeding. The subsequent development of rapidly progressive renal failure, active urinary sediment, and purpura revealed an underlying systemic vasculitic process, followed by fatal diffuse alveolar haemorrhage. This case highlights the importance of recognising gastrointestinal manifestations as a potential early feature of systemic vasculitis and of interpreting gastrointestinal, renal, cutaneous, and pulmonary findings as part of a unified clinical picture.

## Case presentation

A 72-year-old woman presented with a one-week history of bloody diarrhoea. It was associated with abdominal pain, vomiting, lethargy, bilateral lower limb oedema, and new purpura over the thighs and soles of the feet. Her medical history included osteoarthritis of the knee only. Four weeks before admission, she had taken a short course of co-amoxiclav and two weeks of daily ibuprofen for ankle pain, stopping the latter when persistent vomiting developed.

On examination, she was pale, peripherally cool, tachypnoeic, and tachycardic. Arterial blood gas showed severe metabolic acidosis (pH: 7.177, HCO₃⁻: 11.7 mmol/L, lactate: 1.1 mmol/L). Initial investigations revealed marked leucocytosis, elevated inflammatory markers, coagulopathy, and severe acute kidney injury (AKI), with an estimated glomerular filtration rate (eGFR) of 4 mL/min/1.73 m² against a baseline of 82 mL/min/1.73 m² (Table 1).

**Table 1:** Initial laboratory investigations on admission The table summarises the patient's admission laboratory results, with upward (↑) and downward (↓) arrows indicating values above and below the reference ranges, respectively. Reference ranges are provided for comparison. ALP: alkaline phosphatase, ALT: alanine aminotransferase, CRP: C-reactive protein, INR: international normalised ratio

Parameter	Result	Reference range
CRP	136 mg/L (↑)	0-10 mg/L
White blood cell count	32.9 × 10⁹/L (↑)	4-11 × 10⁹/L
Neutrophils	32.40 × 10⁹/L (↑)	1.50-7.50 × 10⁹/L
Haemoglobin	97 g/L (↓)	115-165 g/L
Reticulocytes	161 × 10⁹/L (↑)	20-80 × 10⁹/L
Platelets	233 × 10⁹/L	140-400 × 10⁹/L
Sodium	131 mmol/L	133-146 mmol/L
Potassium	3.9 mmol/L	3.5-5.3 mmol/L
Urea	41.8 mmol/L (↑)	2.5-7.8 mmol/L
Creatinine	761 µmol/L (↑)	60-120 µmol/L
Bilirubin	37 µmol/L (↑)	3-17 µmol/L
ALT	33 U/L	7-56 U/L
ALP	183 U/L (↑)	30-130 U/L
Albumin	28 g/L	35-50 g/L
INR	2.1 (↑)	0.8-1.2
Fibrinogen	3.2 g/L	2-4 g/L

Urinalysis showed 3+ blood, 2+ protein, a urine protein:creatinine ratio of 450 mg/mmol, and urinary β₂-microglobulin > 18 mg/L. Contrast-enhanced computed tomography (CT) of the abdomen and pelvis demonstrated colitis extending from the rectum to the proximal transverse colon, with additional distal ileal and jejunal enteritis (Figure 1). The initial differential diagnosis included infective, inflammatory, and ischaemic colitis. Faecal PCR testing was sent on admission and was negative for the tested enteric pathogens, including Shiga toxin-producing *Escherichia coli* (STEC). *Clostridioides difficile* testing was also negative. Peripheral blood cultures showed no growth after five days of incubation. These results reduced the likelihood of the tested enteric infections but did not exclude every possible infectious cause of colitis.

**Figure 1:**
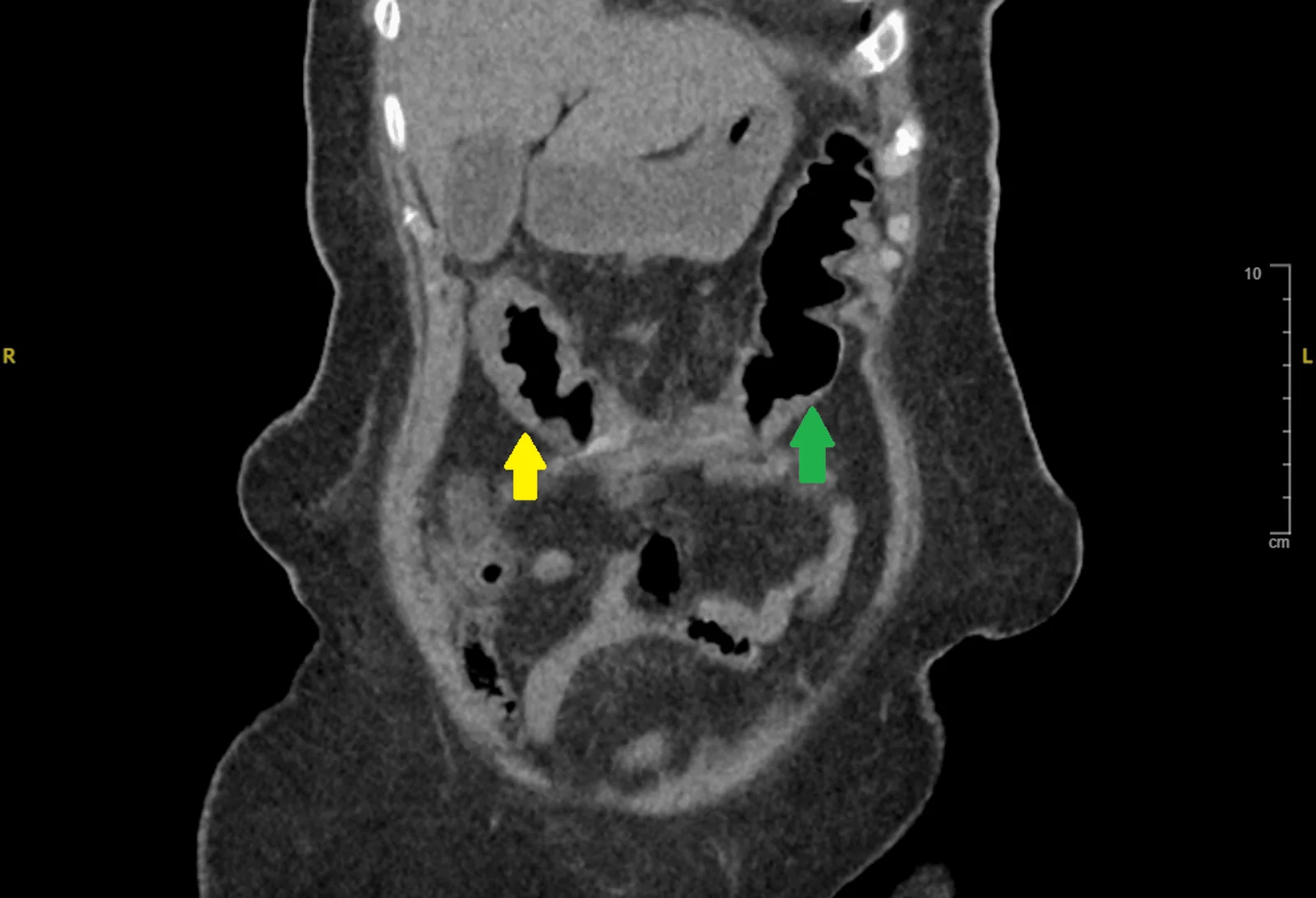
Coronal abdominal CT showing colitis The yellow arrow indicates colonic wall thickening consistent with active colitis in the proximal transverse colon. The green arrow highlights the transition to a normal-appearing colonic wall in the distal transverse colon. CT: computed tomography

After the initial laboratory investigations on admission, an autoimmune screen and renal serology were sent with the initial renal screen. MPO-ANCA returned strongly positive at >221.9 IU/mL by day 2, supporting a diagnosis of MPO-ANCA-associated vasculitis. Anti-glomerular basement membrane (anti-GBM) antibody was negative (<2.9 CU), excluding double-positive disease. She was immediately transferred to the renal team.

The peripheral blood film showed rouleaux formation, polychromasia, burr cells, rare bite cells, and very occasional schistocytes. Although the platelet count was initially preserved at 233 × 10⁹/L, the elevated reticulocyte count, hyperbilirubinaemia, and subsequent fall in haemoglobin raised the possibility of haemolysis. These findings prompted consideration of thrombotic microangiopathy (TMA), including haemolytic uraemic syndrome (HUS). However, in the absence of lactate dehydrogenase (LDH) and haptoglobin results, and given the scarcity of schistocytes and normal initial platelet count, the available evidence was insufficient to establish a diagnosis of TMA or HUS.

Over the second day, her condition worsened with profound metabolic acidosis (pH: 7.04, HCO₃⁻: 9.3 mmol/L) resistant to fluid resuscitation, necessitating ICU review and intravenous (IV) sodium bicarbonate. She exhibited widespread systemic involvement, including severe acute kidney injury, active urinary sediment, proteinuria, purpura, ongoing rectal bleeding, and gross haematuria with haemoglobin dropping to 76 g/L. A renal biopsy was performed during the admission; however, the histopathological report became available only after the patient's death. The subsequent histopathological examination demonstrated necrotising crescentic pauci-immune glomerulonephritis, providing histopathological confirmation of renal involvement by ANCA-associated vasculitis.

At the renal ward, urgent remission-induction therapy was commenced with pulsed intravenous methylprednisolone and intravenous cyclophosphamide. Given the severity of her renal involvement, therapeutic plasma exchange (PLEX) and intermittent haemodialysis were also initiated, alongside blood transfusion. Between days 3 and 6, she underwent three haemodialysis sessions and two PLEX cycles, with subsequent improvement in acid-base status (pH: 7.44, HCO₃⁻: 23.5 mmol/L). However, coagulopathy persisted, with an international normalised ratio (INR) of 1.8 and fibrinogen of 1 g/L, requiring vitamin K, desmopressin, and blood products.

On day 7, she developed worsening bilateral lower limb oedema and bibasal crepitations, followed by two episodes of haemoptysis. By day 8, she had progressed to severe DAH, with profuse oronasal bleeding, gross haemoptysis, tachypnoea, tachycardia, and hypoxia refractory to high-flow oxygen therapy. Her haemoglobin fell from 51 to 36 g/L, with worsening thrombocytopenia (platelets: 52 × 10⁹/L), persistent coagulopathy (INR: 1.8), and recurrent severe acidaemia (pH: 6.983, HCO₃⁻: 10.6 mmol/L). Chest radiography demonstrated extensive bilateral pulmonary haemorrhage (Figure 2).

**Figure 2:**
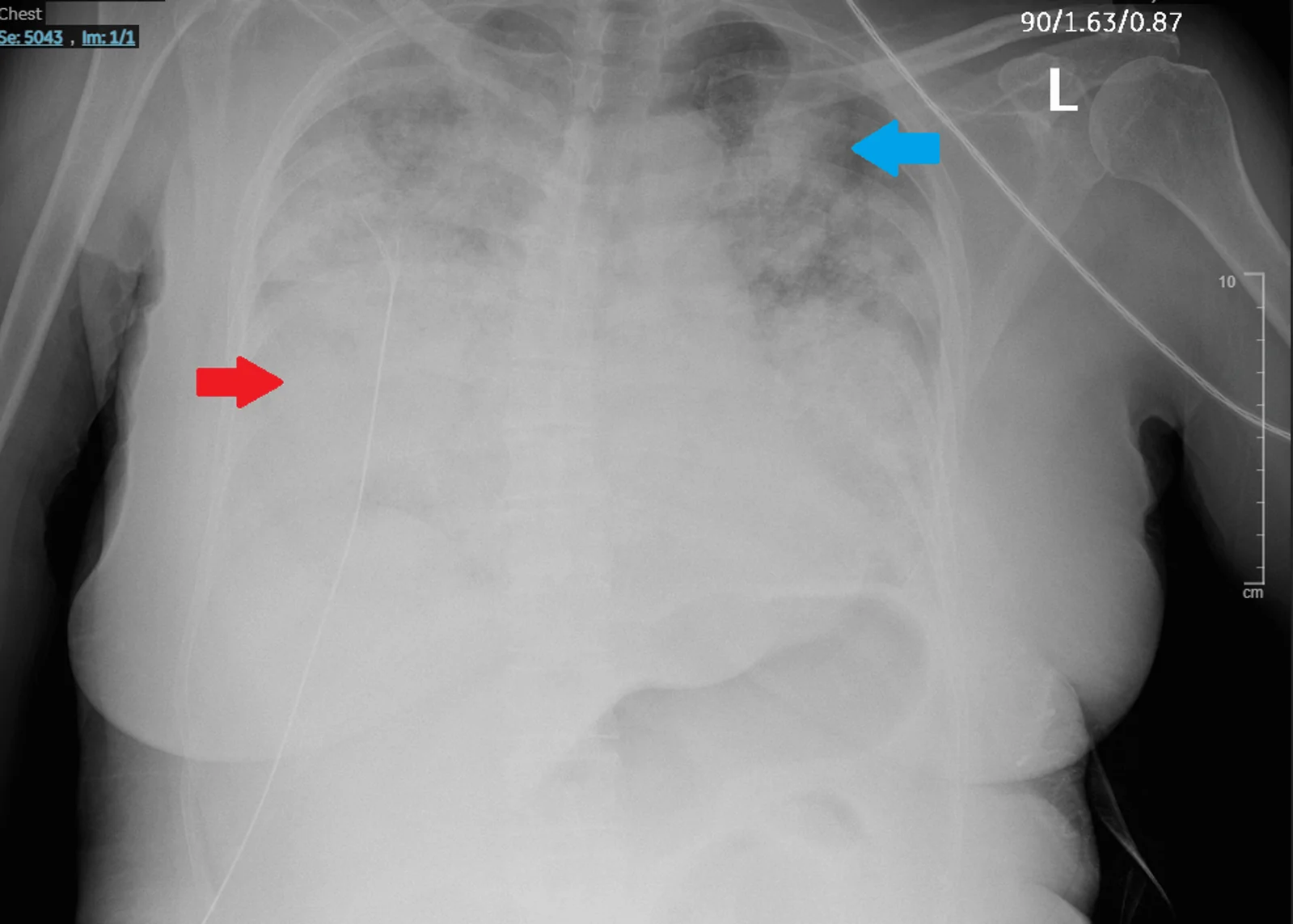
Portable AP chest radiograph demonstrating diffuse pulmonary haemorrhage secondary to vasculitis The red arrow indicates extensive bilateral pulmonary airspace opacities consistent with diffuse alveolar haemorrhage. The blue arrow highlights relative sparing of the peripheral lung fields. AP: anteroposterior

A massive haemorrhage protocol was activated with rapid transfusion of packed red cells, fresh frozen plasma, and platelets. Following urgent multidisciplinary review, invasive ventilation was deemed unlikely to alter the outcome given irreversible multi-organ failure, refractory acidaemia, and consumptive coagulopathy. A do not attempt cardiopulmonary resuscitation (DNACPR) order with a ward-based ceiling of care was instituted, and care was transitioned to palliation. The patient died shortly afterward.

## Discussion

This case illustrates a fulminant presentation of MPO-ANCA-associated vasculitis (AAV), with a clinical phenotype consistent with microscopic polyangiitis (MPA), in which gastrointestinal inflammation and bleeding, severe renal involvement, cutaneous purpura, and pulmonary haemorrhage evolved over several days into fatal multi-organ failure. The diagnostic challenge was that gastrointestinal symptoms dominated the initial presentation, closely mimicking primary gastrointestinal disease. Although the subsequent combination of haematuria, proteinuria, rapidly progressive acute kidney injury (AKI), purpura, strongly positive MPO-ANCA, and biopsy-confirmed pauci-immune glomerulonephritis established systemic AAV, the cause of the gastrointestinal abnormalities remained uncertain. Intestinal vasculitis was not confirmed histologically, and ischaemic colitis secondary to hypoperfusion cannot be excluded. This case highlights the importance of recognising systemic features that may indicate AAV in patients presenting with severe gastrointestinal illness and otherwise unexplained renal dysfunction.

Gastrointestinal involvement in AAV is less common than renal and pulmonary disease, although reported frequencies vary between studies and disease phenotypes [5,6]. Gastrointestinal symptoms are often non-specific, and intestinal vasculitis may only be recognised following severe complications such as bleeding, infarction, or perforation [5,6]. In a dedicated series of vasculitic gastrointestinal disease, patients with peritonitis, bowel perforation, or intestinal infarction had six-month and five-year survival rates of 60% and 46%, respectively [5], suggesting that severe gastrointestinal complications identify a particularly high-risk clinical presentation.

In this case, CT demonstrated extensive colitis and additional small bowel enteritis, accompanied by gastrointestinal bleeding. However, gastrointestinal biopsy and mesenteric angiography were not performed because of haemodynamic instability, active bleeding, and persistent coagulopathy. Consequently, direct evidence of intestinal vasculitis was unavailable. Furthermore, a notable limitation in evaluating potential microangiopathic haemolysis in this setting was the lack of available serum lactate dehydrogenase (LDH) and haptoglobin levels. Although the gastrointestinal findings occurred alongside strongly positive MPO-ANCA and biopsy-confirmed renal AAV, a causal relationship between the vasculitis and bowel pathology cannot be established. Ischaemic colitis secondary to hypoperfusion, potentially compounded by systemic inflammation and critical illness, remains a plausible alternative explanation. The gastrointestinal manifestations should therefore be interpreted in the context of systemic AAV without assuming that direct intestinal vasculitis was definitively present.

The renal phenotype was particularly severe, with an eGFR of 4 mL/min/1.73 m² on admission. Although gastrointestinal fluid losses may have contributed to renal hypoperfusion, the active urinary sediment, haematuria, proteinuria, and severity of AKI suggested intrinsic glomerular disease. A renal biopsy was performed during the admission, with the histopathological report becoming available after the patient's death. The biopsy demonstrated necrotising crescentic pauci-immune glomerulonephritis, providing histopathological confirmation of renal involvement by ANCA-associated vasculitis in the context of strongly positive MPO-ANCA [1,3,4]. Urinary β₂-microglobulin, a marker of proximal tubular reabsorptive capacity, was also markedly elevated, indicating tubular dysfunction, although this is non-specific and should be regarded as supportive evidence of tubular injury rather than a diagnostic marker of AAV [7]. This case emphasises that severe AKI accompanying gastrointestinal illness should not automatically be attributed to volume depletion.

MPO-AAV results from loss of immune tolerance to myeloperoxidase, influenced by genetic susceptibility and environmental factors [1,3]. Following inflammatory priming, ANCA binding activates neutrophils, promoting degranulation, reactive oxygen species production, and neutrophil extracellular trap formation, with amplification through the alternative complement pathway and C5a-mediated neutrophil recruitment [3,8]. This mechanism can result in glomerular, mesenteric, cutaneous, and pulmonary vascular injury. Her recent ibuprofen exposure is more appropriately regarded as a potential aggravating factor than the primary cause of the vasculitis. Non-steroidal anti-inflammatory drugs (NSAIDs) can impair renal perfusion through reduced prostaglandin-mediated afferent arteriolar vasodilation [9]. This differs from classical drug-induced AAV, which is more strongly associated with agents such as hydralazine and propylthiouracil [1,3].

The revised Five-Factor Score (FFS) provides prognostic stratification based on age > 65 years, cardiac insufficiency, gastrointestinal involvement, renal insufficiency, and, in granulomatosis with polyangiitis and eosinophilic granulomatosis with polyangiitis, absence of ENT manifestations. Five-year mortality in the derivation cohort was 9%, 21%, and 40% for scores of 0, 1, and ≥2, respectively [10]. This patient had three applicable risk factors: age > 65 years, renal insufficiency, and gastrointestinal involvement. Although the FFS is not designed to predict individual outcomes, these features indicate a high-risk clinical phenotype. Her subsequent diffuse alveolar haemorrhage, profound anaemia, refractory metabolic acidaemia, thrombocytopenia, and consumptive coagulopathy represented additional markers of rapidly progressive, life-threatening disease.

DAH is a recognised severe manifestation of AAV caused by pulmonary capillaritis and disruption of the alveolar-capillary barrier [3,4]. In this patient, haemoptysis, falling haemoglobin, refractory hypoxaemia, and bilateral pulmonary haemorrhage developed rapidly after her initial presentation. The combination of ongoing pulmonary bleeding with thrombocytopenia and coagulopathy further compromised haemostasis and oxygen delivery. Once multi-organ failure has developed, even aggressive immunosuppression and organ support may be insufficient to alter the outcome, highlighting the narrow therapeutic window of fulminant AAV.

Despite the administration of pulsed methylprednisolone, cyclophosphamide, plasma exchange, and renal replacement therapy, multi-organ failure progressed quickly. Current guidance supports high-dose glucocorticoids in combination with cyclophosphamide or rituximab for organ-threatening AAV [11].

The role of plasma exchange in AAV is more nuanced following the PEXIVAS trial, which found no significant reduction in death or end-stage kidney disease with plasma exchange added to standard therapy in severe AAV [12]. Current guidance supports consideration of plasma exchange in selected patients with severe renal involvement, particularly those requiring dialysis or with rapidly progressive renal dysfunction, while its use in hypoxaemic diffuse alveolar haemorrhage may also be considered [11,13,14]. In this patient, plasma exchange was initiated because of severe renal involvement requiring dialysis, before the subsequent development of diffuse alveolar haemorrhage. Her deterioration despite treatment illustrates the limitations of rescue therapy once extensive multi-organ injury has developed.

## Conclusions

This case demonstrates an unusually aggressive presentation of MPO-ANCA-associated vasculitis, with biopsy-confirmed necrotising crescentic pauci-immune glomerulonephritis, cutaneous purpura, severe gastrointestinal inflammation and bleeding, and subsequent fatal diffuse alveolar haemorrhage. The cause of the gastrointestinal abnormalities remained uncertain, as intestinal vasculitis was not histologically confirmed and ischaemic injury secondary to hypoperfusion could not be excluded. Recent NSAID exposure and the initial clinical resemblance to infective or ischaemic colitis further complicated the diagnostic picture.

The key clinical lesson is that severe gastrointestinal illness accompanied by unexplained renal dysfunction, haematuria, proteinuria, or purpura should prompt consideration of systemic AAV. Although the gastrointestinal findings in this case cannot be definitively attributed to vasculitis, their coexistence with rapidly progressive glomerulonephritis illustrates the importance of recognising a potentially systemic disease process. Despite prompt immunosuppression, plasma exchange, and renal replacement therapy, the patient developed fatal diffuse alveolar haemorrhage, highlighting the potential for rapid deterioration once severe multi-organ injury has occurred.
